# Dephosphorylation of the Core Septin, AspB, in a Protein Phosphatase 2A-Dependent Manner Impacts Its Localization and Function in the Fungal Pathogen *Aspergillus fumigatus*

**DOI:** 10.3389/fmicb.2016.00997

**Published:** 2016-06-22

**Authors:** José M. Vargas-Muñiz, Hilary Renshaw, Amber D. Richards, Greg Waitt, Erik J. Soderblom, Martin. A. Moseley, Yohannes Asfaw, Praveen R. Juvvadi, William J. Steinbach

**Affiliations:** ^1^Department of Molecular Genetics and Microbiology, Duke University Medical Center, DurhamNC, USA; ^2^Division of Pediatric Infectious Diseases, Department of Pediatrics, Duke University Medical Center, DurhamNC, USA; ^3^Duke Proteomics and Metabolomics Core Facility, Center for Genomic and Computational Biology, Duke University, DurhamNC, USA; ^4^Department of Laboratory Animal Resources, Duke University Medical Center, DurhamNC, USA

**Keywords:** *Aspergillus fumigatus*, septin, phosphorylation, kinase, phosphatase

## Abstract

Septins are a conserved family of GTPases that form hetero–oligomeric complexes and perform diverse functions in higher eukaryotes, excluding plants. Our previous studies in the human fungal pathogen *Aspergillus fumigatus* revealed that the core septin, AspB, a CDC3 ortholog, is required for septation, conidiation, and conidial cell wall organization. Although AspB is important for these cellular functions, nothing is known about the role of kinases or phosphatases in the posttranslational regulation and localization of septins in *A. fumigatus*. In this study, we assessed the function of the Gin4 and Cla4 kinases and the PP2A regulatory subunit ParA, in the regulation of AspB using genetic and phosphoproteomic approaches. Gene deletion analyses revealed that Cla4 and ParA are indispensable for hyphal extension, and Gin4, Cla4, and ParA are each required for conidiation and normal septation. While deletion of *gin4* resulted in larger interseptal distances and hypervirulence, a phenotype mimicking *aspB* deletion, deletion of *cla4* and *parA* caused hyperseptation without impacting virulence, indicating divergent roles in regulating septation. Phosphoproteomic analyses revealed that AspB is phosphorylated at five residues in the GTPase domain (S134, S137, S247, T297, and T301) and two residues at its C-terminus (S416 and S461) in the wild-type, Δ*gin4* and Δ*cla4* strains. However, concomitant with the differential localization pattern of AspB and hyperseptation in the Δ*parA* strain, AspB remained phosphorylated at two additional residues, T68 in the N-terminal polybasic region and S447 in the coiled-coil domain. Generation of nonphosphorylatable and phosphomimetic strains surrounding each differentially phosphorylated residue revealed that only AspB*^mt^*-T68E showed increased interseptal distances, suggesting that dephosphorylation of T68 is important for proper septation. This study highlights the importance of septin phosphorylation/dephosphorylation in the regulation of *A. fumigatus* hyphal septation.

## Introduction

Septins, a conserved family of GTPases, are involved in a variety of critical cellular functions, ranging from cell division to cell wall maintenance (Momany et al., 2001; Alvarez-Tabares and Perez-Martin, 2010; Kozubowski and Heitman, 2010; Lindsey et al., 2010; Hernandez-Rodriguez et al., 2012, 2014). The number of septin-encoding genes varies amongst all organisms. In the pathogenic fungus *Candida albicans* there are seven septin genes, all of which have orthologs in the model yeast *Saccharomyces cerevisiae*, while *Aspergillus fumigatus* has only five septin genes (Warenda and Konopka, 2002; Pan et al., 2007; Juvvadi et al., 2011b). *A. fumigatus aspA, aspB, aspC*, and *aspD* are orthologous to *S. cerevisiae* *CDC11, CDC3, CDC12*, and *CDC10*, respectively; while *aspE* is absent in *S. cerevisiae* (Pan et al., 2007; Lindsey et al., 2010; Juvvadi et al., 2011b). Deletion analyses in *A. fumigatus* revealed that none of the septins are required for growth under basal conditions, yet septins AspA, AspB, AspC, and AspE play a key role in regular septation and AspA, AspB, AspC, and AspD regulate conidiation (Vargas-Muniz et al., 2015). Deletion of *aspB* resulted in hypervirulence in an invertebrate model of invasive aspergillosis, as well as increased susceptibility and AspB mislocalization following exposure to anti-cell wall agents.

Septins function through formation of heteropolymeric complexes; however, regulation of the formation of these complexes is unclear (Gladfelter, 2010). Multiple lines of evidence suggest that posttranslational modification, such as phosphorylation, regulates the formation, and stability of septin complexes. For example, posttranslational modification of septins in *S. cerevisiae* is key in controlling the assembly of septins into higher-order structures (Tang and Reed, 2002; Garcia et al., 2011). Septin Cdc3, Cdc10, Cdc11, Cdc12, and Shs1 are also phosphorylated in the filamentous hemiascomycete *Ashbya gossypii* (Meseroll et al., 2013). Phosphomimetic mutation of *A. gossypii* septin Cdc12 and Shs1 phosphorylation sites resulted in lethality, supporting the notion of septin phosphorylation as a key regulator of proper septin function (Meseroll et al., 2013).

Kinases Gin4, and Cla4 have been shown to phosphorylate septins in hemiascomycetes (Longtine et al., 1998; Kadota et al., 2004; Versele and Thorner, 2004; Wightman et al., 2004; Sinha et al., 2007; DeMay et al., 2009; Li et al., 2012). In *S. cerevisiae*, Gin4 co-purifies with and is regulated by the septins (Barral et al., 1998; Mortensen et al., 2002). Gin4 requires this interaction with the septin complex in order to be activated by hyperphosphorylation and subsequently phosphorylates the non-core septin SHS1 (Barral et al., 1998; Mortensen et al., 2002). This co-dependence between Gin4 and septins has also been demonstrated in *C. albicans*, where deletion of *gin4* eliminates septin ring formation (Wightman et al., 2004). Another important regulator of septins in *S. cerevisiae* is the PAK kinase Cla4. In the *S. cerevisiae*Δ*cla4* strain, septins form a band at the tip of the bud instead of localizing at the bud neck (Cvrekova et al., 1995). Furthermore, Cla4 is capable of interacting *in vitro* with and phosphorylating septins Cdc3 and Cdc10. Cla4 phosphorylates Cdc10 on two serine residues (S256 and S312), and alanine substitution of serine 256 resulted in an elongated bud at 37°C, indicating that this phosphorylation site is required for proper Cdc10 function (Versele and Thorner, 2004). Although the essential roles of Gin4 and Cla4 in septin phosphorylation have been described in hemiascomycetes, their role as potential septin regulators has not been explored in filamentous ascomycetes. Recent work in the model filamentous fungi *Neurospora crassa* and *A. nidulans* has shown that Cla4 is involved in hyphal extension, polarity, and asexual and sexual reproduction (Park et al., 2011; De Souza et al., 2013). The filamentous plant pathogen *Magnaporthe oryzae cla4* ortholog (CHM1) is also involved in hyphal extension, polarized growth, new growth foci limitation, conidiation and pathogenesis (Li et al., 2004). In the basidiomycete *Ustilago maydis, cla4* is involved in budding, cytokinesis, cell wall assembly, mating and pathogenesis (Leveleki et al., 2004).

Although some septin kinases have been explored, less is known of septin phosphatases. In *S. cerevisiae*, Rts1, a protein phosphatase 2A (PP2A) subunit, regulates septin dephosphorylation during telophase, and this dephosphorylation contributes to cytokinesis (Dobbelaere et al., 2003). In *A. nidulans*, deletion of *parA* (*RTS1* ortholog) resulted in hyperseptation and reduction in conidiation; however, the possible septin regulatory role was not defined (Zhong et al., 2014).

In this study, we built on our previous explorations of septins in the pathogen *A. fumigatus* and defined for the first time the phosphorylation status of the core septin AspB and the role of Gin4, Cla4, and ParA as AspB regulators in *A. fumigatus* growth and development. We found that ParA and Cla4 are indispensable for full hyphal extension, whereas Gin4, Cla4, and ParA are important for conidiation and required for proper localization of AspB. While deletion of *gin4* resulted in increased interseptal distances, deletion of *cla4* or *parA* resulted in hyperseptation in more basal compartments. Similar to the Δ*aspB* strain, the Δ*gin4* strain is hypervirulent in the *Galleria mellonella* invertebrate and murine models of invasive aspergillosis. Phosphoproteomic analyses revealed that AspB is phosphorylated in seven residues, five of which are located in the GTPase domain. Interestingly, although the deletion of kinases *gin4* or *cla4* did not affect the phosphorylation status of AspB, the deletion of the phosphatase subunit *parA* resulted in the phosphorylation of two additional sites, including one residue (S447) in the coiled-coil domain. Mutation of these residues to alanine (blocking phosphorylation) or glutamic acid (phosphomimetic) mislocalized AspB and caused an increase in interseptal distances in the AspB*^mt^-*T68E strain. Taken together, these important findings suggest that ParA is a potential direct regulator of AspB through dephosphorylation, while Cla4 and Gin4 could regulate AspB through a yet unknown indirect mechanism.

## Materials and Methods

### Strains, Media, and Culture Conditions

The *A. fumigatus akuB*^KU80^ *pyrG^-^* uracil/uridine auxotroph was used for deletion analyses and the *akuB*^KU80^ uracil/uridine prototroph was used as the wild-type strain (da Silva Ferreira et al., 2006; **Table 1**). Cultures were grown on glucose minimal media (GMM) at 37°C, except where otherwise specified. *Escherichia coli* DH5α competent cells were used for cloning.

**Table 1:** Strains used in the present study.

Strain	Parent strain	Genotype	Reference on source
*akuB*^KU80^ *pyrG^-^*	CEA17 *pyrG^+^*	*pyrG*	da Silva Ferreira et al., 2006
*akuB*^KU80^	CEA17	Wild-type	da Silva Ferreira et al., 2006
*aspB–egfp*	*akuB*^KU80^	*ΔaspB::aspBpromo-aspB–egfp-hph*	Vargas-Muniz et al., 2015
*Δgin4*	*akuB*^KU80^ *pyrG^-^*	*Δgin4::pyrG*	This study
*Δcla4*	*akuB*^KU80^ *pyrG^-^*	*Δcla4::pyrG*	This study
*ΔparA*	*akuB*^KU80^ *pyrG^-^*	*ΔparA::pyrG*	This study
*Δgin4 ΔaspB*	*Δgin4*	*Δgin4::pyrG ΔaspB::ble*	This study
*Δgin4 aspB–egfp*	*Δgin4*	*Δgin4::pyrG ΔaspB::aspBpromo-aspB–egfp-hph*	This study
*Δcla4 aspB–egfp*	*Δcla4*	*Δcla4::pyrG ΔaspB::aspBpromo-aspB–egfp-hph*	This study
*ΔparA aspB–egfp*	*ΔparA*	*ΔparA::pyrGΔaspB::aspBpromo-aspB–egfp-hph*	This study
*aspB^mt^-S447A*	*akuB*^KU80^	*ΔaspB::aspBpromo- aspB^mt^-S447A -egfp-hph*	This study
*aspB^mt^-S447E*	*akuB*^KU80^	*ΔaspB::aspBpromo- aspB^mt^-S447E -egfp-hph*	This study
*aspB^mt^-T68A*	*akuB*^KU80^	*ΔaspB::aspBpromo- aspB^mt^-T68A -egfp-hph*	This study
*aspB^mt^-T68E*	*akuB*^KU80^	*ΔaspB::aspBpromo- aspB^mt^-T68E -egfp-hph*	This study

### Construction of Septin Deletion Strains

Deletion of *cla4* was performed by replacing the 2.6 kb *cla4* gene (Afu5g05900)^1^ with the 2.4 kb *pyrG* gene from *A. parasiticus*. Approximately 1 kb of upstream and downstream flanking regions of *cla4* were PCR-amplified from AF293 genomic DNA (Supplementary Table S1). *pyrG* was amplified from the pJW24 plasmid. The *cla4* deletion construct was generated by fusion PCR and transformed into the *akuB*^KU80^ *pyrG^-^* strain, as previously described (Steinbach et al., 2006). Deletion of *gin4* was performed by similarly replacing the 4.0 kb *gin4* gene (Afu6g02300) with the 2.4 kb *pyrG* gene from *A. parasiticus.* Deletion of *parA* was attained by replacing the 2.5 kb *parA* gene (Afu5g02560) with the 3.0 kb *A. parasiticus pyrG* cassette, and the resulting plasmid was digested with NotI and SalI and transformed into *akuB*^KU80^ *pyrG^-^*. Each deletion strain was then transformed with the *aspB–egfp* construct as previously described (Vargas-Muniz et al., 2015). All gene deletions were confirmed by both PCR (data not shown) and Southern analyses (Supplementary Figure S1).

### Radial Growth and Conidial Quantification

Conidia (10^4^) from each strain were inoculated on GMM agar, incubated at 37°C, and radial growth measured every 24 h for 5 days. For conidial quantification, 10^4^ conidia from each strain were inoculated on GMM agar, incubated for 5 days at 37°C, and conidia harvested in 10 ml of 0.05% Tween-80 and quantified using a hemocytometer as previously described (Lamoth et al., 2012). All assays were performed in triplicate. Student’s t-tests were performed using Graph Pad Prism (San Diego, CA, USA).

### Aniline Blue Staining

Aniline blue stain was used for detection of cell wall β-glucan. 10^4^ conidia of each strain were cultured on coverslips immersed in 10 ml of GMM + UU broth and incubated for 20 h at 37°C, as previously described (Juvvadi et al., 2011a). The coverslips were rinsed with GMM + UU broth, inverted over 500 μl of aniline blue stain, and incubated for 5 min at 25°C. Coverslips were rinsed once more briefly with GMM + UU broth and observed by fluorescence microscopy.

### *Galleria mellonella* and Murine Invasive Aspergillosis Virulence Models

Virulence of the deletion strains was assessed in an iterative fashion using an invertebrate and then a murine model of invasive aspergillosis. For the initial invertebrate model, 20 larvae of the wax moth *G. mellonella* were infected with each of the *A. fumigatus* deletion strains or the wild-type strain, delivering 5 μl of a 1 × 10^8^ conidia/ml of suspension with a total inoculum size of 2 × 10^5^ conidia. Infected larvae were incubated at 37°C and survival scored daily for 5 days (Steinbach et al., 2006). For the murine model, male mice (CD1, Charles River Laboratory, Raleigh, NC, USA) were immunosuppressed with cyclophosphamide (175 mg/kg, intraperitoneally, days -2 and +3) and triamcinolone acetonide (40 mg/kg, subcutaneously, days -1 and +6). A total inoculum of 4 × 10^6^ conidia was delivered intranasally using 40 μl of 10^8^ conidia of the Δ*gin4*, Δ*cla4*, Δ*parA*, and *akuB*^KU80^ strains (Vargas-Muniz et al., 2015). Survival for both virulence models was plotted on a Kaplan–Meier curve and analyzed using log rank pair-wise comparison. Animal studies were carried out in accordance with all of the guidelines of the Duke University Medical Center Institutional Animal Care and Use Committee (IACUC) and in compliance with the United States Animal Welfare Act (Public Law 98-198). Duke University Medical Center IACUC approved all of the vertebrate studies. The studies were conducted in the Division of Laboratory Animal Resources (DLAR) facilities that are accredited by the Association for Assessment and Accreditation of Laboratory Animal Care (AAALAC).

### Histopathology Analyses

To characterize *in vivo* disease histopathology, three additional mice per each single deletion strain were infected. Mice were euthanized on day +3 after inoculation and lungs were harvested. Lungs sections were stained with Gomori’s methenamine silver stain to stain fungal hyphae and hematoxylin and eosin stain to examine inflammation (Steinbach et al., 2006).

### Fluorescence Microscopy

Conidia (10^4^) of each *aspB–egfp* expressing strain were cultured on coverslips immersed in 10 ml of GMM broth and incubated for 20 h at 37°C, as previously described (Vargas-Muniz et al., 2015). Localization patterns were visualized using an Axioskop 2 plus microscope (Zeiss) equipped with AxioVision 4.6 imaging software.

### Protein Extraction, AspB–EGFP Fusion Protein Purification and LC–MS/MS Analysis

Each *A. fumigatus* strain expressing the *aspB–egfp* fusion construct under the control of the *aspB* native promoter was grown in GMM broth for 24 h at 37°C. Biological replicates were prepared for each strain. Total cell lysate was obtained by homogenizing mycelia (600–650 mg wet weight) as previously described (Juvvadi et al., 2013). Total protein in the crude extract was quantified by the Bradford method and normalized to contain 10 mg of protein in the sample before purification using GFP-Trap^®^ affinity purification (Chromotek). GFP-Trap^®^ resin was equilibrated according to the manufacturer’s instructions and resuspended in 100 μl ice-cold dilution buffer (10 mM Tris–HCl pH 7.5, 150 mM NaCl, 0.5 mM EDTA, 1 mM PMSF, 1:100 protease inhibitor cocktail). GFP-Trap^®^ resin suspension was then added to the crude lysate containing the 10 mg of protein and incubated for 2 h at 4°C with gentle agitation. The suspension was centrifuged at 2000 rpm for 10 min at 4°C and the GFP-Trap^®^ pellet was washed once with 500 μl of iced-cold dilution buffer and twice with 500 μl of wash buffer (10 mM Tris–HCl pH 7.5, 350 mM NaCl, 0.5 mM EDTA, 1 mM PMSF, 1:100 protease inhibitor cocktail). The resin was finally washed 3 times with 200 μl of 50 mM ammonium bicarbonate and resuspended in 30 μl of 50 mM ammonium bicarbonate.

Protein bound GFP-Trap^®^ resins suspended in 30 μl 50 mM ammonium bicarbonate, pH 8.0, were supplemented with 0.1% Rapigest SF surfactant (Waters Corp). Samples were reduced with 5 mM dithiolthreitol for 30 min at 70°C and free sulfhydryls were alkylated with 10 mM iodoacetamide for 45 min at room temperature. Proteolytic digestion was accomplished by the addition of 500 ng sequencing grade trypsin (Promega) directly to the resin with incubation at 37°C for 18 h. Supernatants were collected following a 2 min centrifugation at 1,000 rpm, acidified to pH 2.5 with TFA and incubated at 60°C for 1 h to hydrolyze remaining Rapigest surfactant. Insoluble hydrolyzed surfactant was cleared by centrifugation at 15,000 rpm for 5 min. The sample was lyophilized to dryness and phosphopeptides were enriched using GL Biosciences p10 TiO_2_ derivatized tips according to manufacturer protocols. Extracted peptides were lyophilized to dryness and resuspended in 12 μL of 0.2% formic acid/2% acetonitrile.

Each sample was subjected to chromatographic separation on a Waters NanoAquity UPLC equipped with a 1.7 μm BEH130 C_18_ 75 μm I.D. × 250 mm reversed-phase column. The mobile phase consisted of (A) 0.1% formic acid in water and (B) 0.1% formic acid in acetonitrile. Following a 4 μL injection, peptides were trapped for 3 min on a 5 μm Symmetry C_18_ 180 μm I.D. X 20 mm column at 5 μl/min in 99.9% A. The analytical column was then switched in-line and a linear elution gradient of 5% B to 40% B was performed over 30 min at 400 nL/min. The analytical column was connected to a fused silica PicoTip emitter (New Objective, Cambridge, MA, USA) with a 10 μm tip orifice and coupled to a QExactive Plus mass spectrometer through an electrospray interface operating in a data-dependent mode of acquisition. The instrument was set to acquire a precursor MS scan from *m/z* 375–1675 with MS/MS spectra acquired for the ten most abundant precursor ions. For all experiments, HCD (higher energy collisional dissociation) energy settings were 27 v and a 120 s dynamic exclusion was employed for previously fragmented precursor ions.

Raw LC–MS/MS data files were processed in Proteome Discoverer (Thermo Scientific) and then submitted to independent Mascot searches (Matrix Science) against a RefSeq *Aspergillus* database containing both forward and reverse entries of each protein. Search tolerances were 5 ppm for precursor ions and 0.02 Da for product ions using trypsin specificity with up to two missed cleavages. Carbamidomethylation (+57.0214 Da on C) was set as a fixed modification, whereas oxidation (+15.9949 Da on M), deamidation (+0.98 Da on NQ), and phosphorylation (+79.99 Da on STY) were considered dynamic mass modifications. All searched spectra were imported into Scaffold (v4.3, Proteome Software) and scoring thresholds were set to achieve a peptide false discovery rate of 1% using the PeptideProphet algorithm. Peak area calculations from extracted ion chromatograms were generated within Skyline v3.5 (MacCoss Lab, University of Washington) following manual peak integration based on identification of retention time and accurate mass.

## Results

### Cla4 and ParA Are Required for Hyphal Extension

**FIGURE 1:**
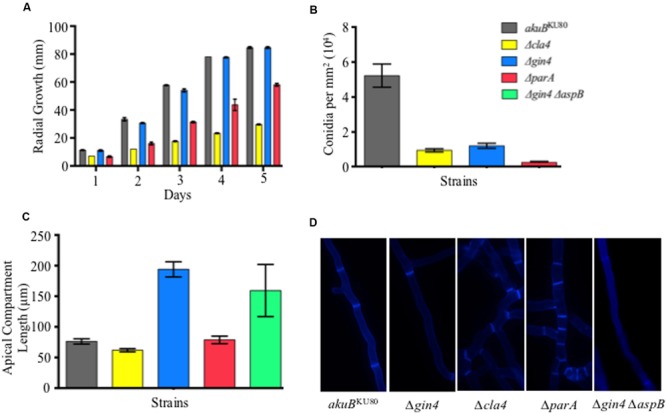
**Septin regulators are required for hyphal extension, conidiation, and proper septation. (A)** Deletion of *parA* and *cla4* results in a 1.5- and 2.9-fold reduction in radial growth, respectively (*p* < 0.001). Conidia (10^4^) from each septin deletion strain and wild-type strain were inoculated on glucose minimum media (GMM) and incubated for 5 days at 37°C. **(B)** Deletion of *gin4, cla4*, or *parA* genes resulted in a 4.4-, 5.6-, and 20.7-fold reduction in conidia production, respectively (*p* < 0.001). Conidia were harvested after 5 days of growth using 10 ml of 0.05% Tween-80 and the conidial count was normalized by fungal colony area. **(C)** Deletion of *gin4* results in an increase in length of the apical compartments (*p* < 0.001). Apical compartments were measured following aniline blue staining by fluorescent microscopy after 20 h of incubation at 37°C. **(D)** Aniline blue staining revealed that strains lacking Cla4 or ParA are hyperseptated in basal compartments, while the strain lacking Gin4 contained a larger basal compartment compared to the wild-type strain. Error bars represent standard error of the mean.

**FIGURE 2:**
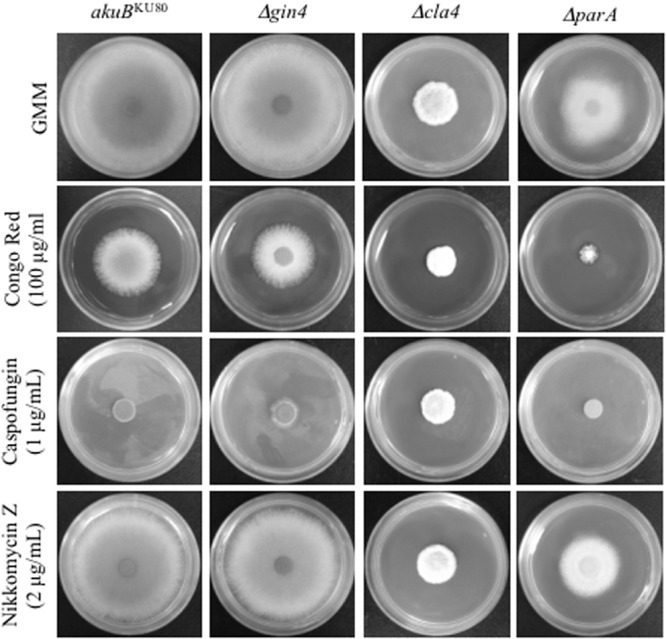
**Deletion of *parA* increased susceptibility to Congo Red, but not to anti-cell wall drugs.** Conidia (10^4^) from each strain were inoculated into GMM agar, GMM agar containing Congo Red (100 μg), Caspofungin (1 μg/ml), or Nikkomycin Z (2 μg/ml), and incubated for 3 days at 37°C.

Our previous studies showed that *A. fumigatus* septins play a pleiotropic role in septation, conidiation, and response to anti-cell wall agents (Vargas-Muniz et al., 2015). In order to gain a further understanding into the regulation and function of AspB in septation, we characterized the possible regulators of AspB using a candidate approach. Based on the literature, we selected the non-essential kinases Gin4 and Cla4, as well as the protein phosphatase 2A (PP2A) subunit ParA, and first defined their contributions to *A. fumigatus* growth and morphology. Deletion of *cla4* or *parA* reduced hyphal extension by 2.9- and 1.5-fold, respectively (*p* < 0.001; **Figure 1A**). Deletion of *aspB* led to an increase in susceptibility to anti-cell wall agents. To test whether the kinases and the PP2A subunit deletion strains phenocopy this increase in susceptibility, the respective deletion strains were cultured in the presence of the cell wall stressor Congo Red as well as the β-glucan synthase inhibitor, caspofungin, and the chitin synthase inhibitor, nikkomycin Z. Only the *ΔparA* strain showed increased susceptibility to Congo Red; however, no increase in susceptibility was noted when the *ΔparA* strain was exposed to anti-cell wall agents in comparison to the *akuB*^KU80^ wild-type strain. (**Figure 2**).

### Gin4, ParA, and Cla4 Are Required for Proper Conidiation and Septation in *A. fumigatus*

Deletion of the *A. fumigatus* core septin AspB resulted in reduction of conidia production and delayed septation (Vargas-Muniz et al., 2015). To further understand the possible role of Cla4, Gin4, and ParA as septin regulators, we measured conidial production in the respective deletion backgrounds (**Figure 1B**). The *Δcla4, Δgin4*, and *ΔparA* strains showed a 5.6-, 4.4-, and 20.7-fold, reduction in conidiation, respectively, compared to the *akuB*^KU80^ wild-type strain (*p* < 0.005). Only the Δ*gin4* strain exhibited a significant increase in apical compartment lengths (2.5-fold, *p* < 0.001; **Figure 1C**). Due to the observed similarity in the apical compartment length between the Δ*gin4* and Δ*aspB* strains, we next generated a Δ*gin4* Δ*aspB* double deletion strain to determine if Gin4 and AspB were acting in the same pathway or were contributing to septation through different mechanisms. The Δ*gin4* Δ*aspB* strain apical compartment length was similar to that of both the Δ*gin4* and Δ*aspB* single deletion strains, indicating that AspB and Gin4 may contribute to septation through the same pathway. In the basal compartments, the Δ*cla4* and Δ*parA* strains exhibited a hyperseptation phenotype, while the Δ*gin4* strain and the Δ*gin4* Δ*aspB* double deletion strain maintained the increased interseptal distances (**Figure 1D**).

### Deletion of *gin4* Results in Hypervirulence in an Invertebrate and a Murine Model of Invasive Aspergillosis

**FIGURE 3:**
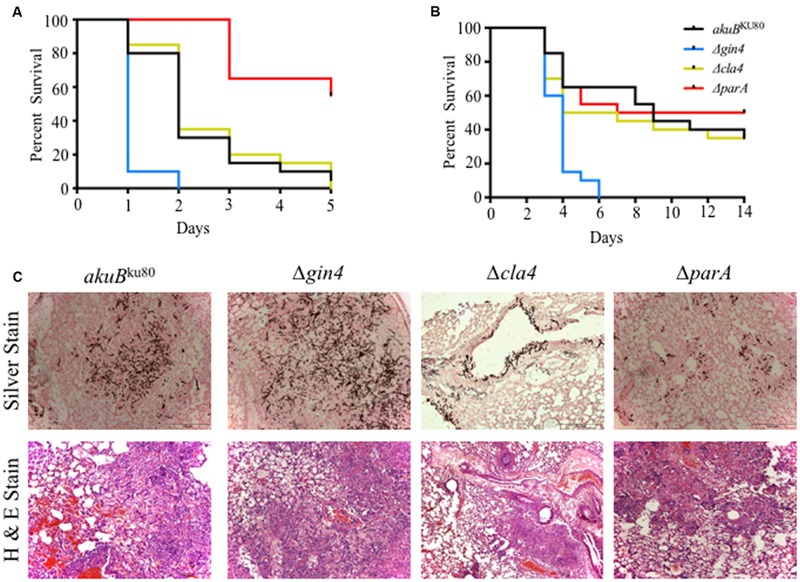
**The Δ*gin4* strain is hypervirulent in both an invertebrate host model and in a persistently immunosuppressed intranasal murine model of invasive aspergillosis. (A)** Infection with the Δ*gin4* strain yield a 100% mortality by day +2 compared to 70% mortality in wild-type strain in the *Galleria mellonella* model (*p* < 0.001). The Δ*parA* strain was hypovirulent (60% survival by day +5) compared to the wild-type strain (*p* < 0.001). A total of 20 waxmoth *G. mellonella* larvae were infected with each *A. fumigatus* strain with 5 μl of a 1 × 10^8^ spores/ml spore suspension. Infected larvae were incubated at 37°C and survival was scored daily for 5 days. **(B)** Deletion of *gin4* results in hypervirulence in a neutropenic murine model of invasive aspergillosis (*p* < 0.001). Mice were infected intranasally with a total of 4 × 10^6^ conidia from each strain and survival monitored for 14 days. **(C)** Lung histopathology performed on day +3 days shows a significant increase in fungal burden (silver staining) and inflammation (H&E staining) between the wild-type and the Δ*gin4* strains.

We previously reported that the Δ*aspB* strain was hypervirulent in the *G. mellonella* model but not in a murine model of invasive aspergillosis. Similar to our findings with the *ΔaspB* strain, the *Δgin4* strain resulted in a significantly increased mortality (100%) in *G. mellonella* by day +3 post infection, compared to the 70% mortality of the *akuB*^KU80^ wild-type strain (*p* < 0.001; **Figure 3A**). In contrast, the *ΔparA* strain had a significantly reduced mortality in this model compared to the wild-type strain, and only 45% mortality at day +5 (*p* < 0.001). The *Δcla4* strain displayed no statistical difference in mortality compared to the wild-type strain (*p* = 0.7971). Building on these invertebrate model virulence results, we then examined these strains in an intranasal murine model of invasive aspergillosis. The Δ*gin4* strain hypervirulence was reproduced in the murine model, with 100% mortality on day +6 (*p* < 0.001), while the Δ*cla4* and Δ*parA* strains were not significantly different from the wild-type strain (50–65% survival; *p* > 0.05; **Figure 3B**). To further understand the contribution of these proteins to pathogenesis, we performed histopathology analysis of the murine lungs after day +3 of infection (**Figure 3C**). Concomitant with the hypervirulence exhibited by the Δ*gin4* strain, there was a significant increase in fungal burden via Gomori’s methenamine silver staining in the wild-type *akuB*^KU80^ and the Δ*gin4* strains. Although the Δ*cla4* and Δ*parA* strain mortality is similar to that of the wild-type strain, they differed in histopathology. The Δ*cla4* strain is defective in tissue invasion and only grew near the alveoli, while the Δ*parA* have a significant reduction in fungal burden. However, the Δ*cla4* and Δ*parA* strains infected mice presented symptoms of infection at the same level as the wild-type strain.

### AspB Exhibits Altered Localization Patterns in the Δ*gin4*,Δ*cla4*, and Δ*parA* Strains

**FIGURE 4:**
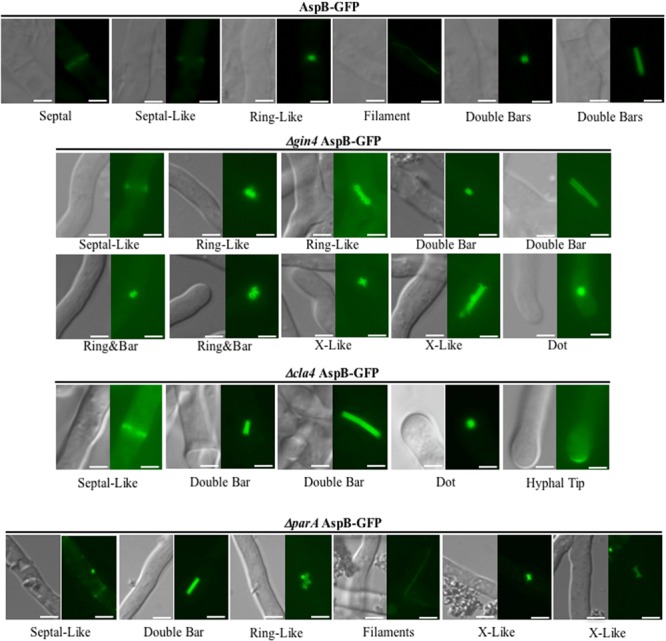
**Deletion of *gin4, cla4*, or *parA* alters AspB localization pattern.** Kinases Gin4 and Cla4, as well as the Protein Phosphatase 2A regulatory sub-unit ParA are required for proper localization of AspB under normal growth condition during the multicellular growth stage. The AspB–EGFP strain was grown in GMM broth for 20 h at 37°C and AspB–EGFP localization visualized by fluorescent microscopy. Scale bar, 2.5 μm.

During the multicellular growth stage, AspB localizes transiently at the septum, at possible septation sites (septal-like), as a ring, and in filaments and double bars (**Figure 4**) (Vargas-Muniz et al., 2015). Here, we found that AspB mislocalized in the *Δgin4*, Δ*cla4*, and Δ*parA* backgrounds (**Figure 4**). In all the strains, including the wild-type strain, AspB localized as septal-like and double bars. In the Δ*parA* strain, the double bars had an intermediate length compared to the wild-type double bar localization. Although AspB in the wild type, *Δgin4*, and *ΔparA* strains localizes to ring-like structures, qualitative observation revealed that the rings in the *Δgin4* and *ΔparA* background strains had a larger diameter compared to the rings observed in the wild-type strain. AspB localized into X-like structures in *Δgin4* and *ΔparA* strains, similar to those observed in the wild-type strain after exposure to caspofungin. While AspB also localized in elongated X-like structures in the *Δgin4* strain, it localized into intermediate length X-like structures in the *ΔparA* strain. In the *Δgin4* and *Δcla4* strains, AspB localized as a dot-like structure near the hyphal tip. Deletion of these kinases abolished the formation of AspB filaments, which remained present in the *ΔparA* strain. AspB in the *Δgin4* strain localized into two different ring and bar structures, one that resembled the ring bar structures observed in the wild-type strain under caspofungin treatment, and another larger ring and bar that is unique to the *Δgin4* strain. AspB localized at the majority of the hyphal tips in the *Δcla4* strain, suggesting that the Cla4 kinase plays a role in excluding AspB from the hyphal tips. The structures described for each strain were commonly found throughout the mycelia. Compartments were observed to contain between 1-2 AspB structures, with the exception of multiple ring-like structures in the Δ*gin4* and *ΔparA* strains that were clumped together.

### AspB Is Differentially Phosphorylated in the *ΔparA* Strain

**FIGURE 5:**
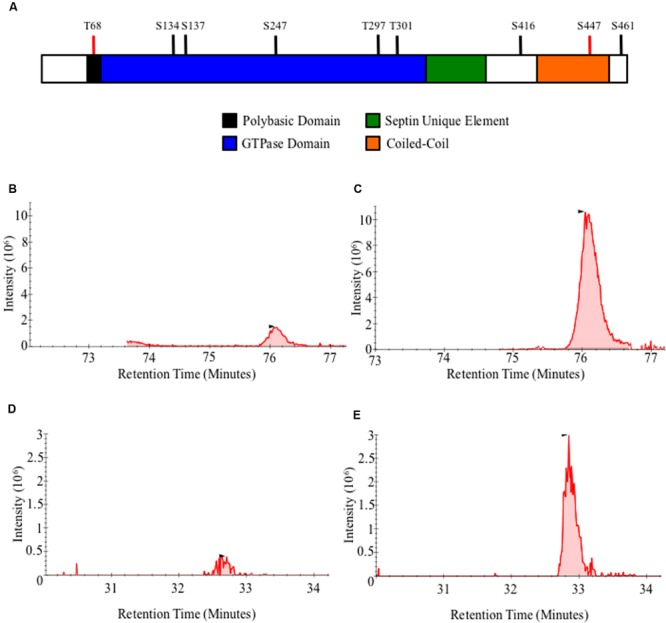
**AspB is differentially phosphorylated in the Δ*parA* strain. (A)** Schematic representation of AspB conserved domains and phosphorylation sites. Black bars indicate AspB phosphorylation sites in the wild-type strain and red bars indicate phosphorylation sites unique to the Δ*parA* strains. **(B–E)** AspB–EGFP and Δ*parA* AspB–EGFP strains were grown in GMM liquid for 24 h at 37°C. AspB–EGFP was purified using GFP-Trap^®^ affinity purification, digested with trypsin and phospho-enriched using TiO_2_. LC–MS/MS was used to identify phosphopeptides. *X*-axis represents intensity of the signal, and *Y*-axis represents the retention time of the phosphopeptide in minutes after mixture injection to the liquid chromatography column. **(B,C)** Chromatogram for the KLTGYVGFANLPNQWHR peptide in **(B)** wild-type and **(C)** Δ*parA* strains. **(C,D)** LESGRPIEEK phosphopeptides chromatogram in **(D)** wild-type and **(E)** Δ*parA* strains. Area under the curve from each chromatogram peak was used for the Skyline analysis.

In order to determine the role of Gin4, Cla4, and ParA as AspB posttranslational regulators, we first determined the phosphorylation profile of AspB in the wild-type strain. AspB was phosphorylated in seven residues: five in the GTPase domain (S134, S137, S247, T297, and T301), and two in the carboxyl-terminus after the septin unique element (S416 and S461) (**Table 2** and **Figure 5A**). We next defined the effect of the deletions of *gin4, cla4*, and *parA* on AspB phosphorylation in a comparative phosphoproteomic approach. This strategy revealed that only the *parA* deletion altered the AspB phosphorylation profile, with AspB phosphorylated at two additional sites: T68 and S447 (**Figures 5B–E**). Quantitative proteomic (Skyline) analyses showed that both Δ*parA* specific phosphoresidues had a sevenfold increase in the area under the curve of the spectrogram compared to the wild-type strain (**Table 3**).

**Table 2:** Phosphorylation sites within AspB identified by TiO_2_ phospho-enrichment followed by LC–MS/MS analysis.

Peptide sequence	Phosphorylated residue	Domain	*m/z*	Mascot ion score
TV[pS]IQSISADIEENGVR	S134	GTPase	82.16 + 0.1	22.4
TVSIQ[pS]ISADIEENGVR	S137	GTPase	87.63 + 0.09	25.2
ADTLTDEEI[pS]LFK	S247	GTPase	82.88 + 0.08	24.3
VPFAVVGAN[pT]EVTTADGR	T297	GTPase	69.93 + 0.08	28.7
VPFAVVGANTEVT[pT]ADGR	T301	GTPase	69.1 + 0.05	21.1
LKQ[pS]EDEKYAR	S416		42.13 + 0.19	43.0
KGF[pS]LR	S461	C-terminus	44.16 + 0.25	33.2

**Table 3:** Differential phosphorylation of AspB in the Δ*parA* strain identified by Skyline Analysis of peaks obtained after TiO_2_ enrichment and LC–MS/MS.

Peptide sequence	Phosphorylated residue	*m/z*	*akuB*^KU80^ Area	Δ *parA* Area	Fold change
KL[pT]GYVGFANLPNQWHR	T68	76.06 ± 0.05	46987743	327294944	6.97
LE[pS]GRPIEEK	S447	32.84 ± 0.12	55234844	8304621.5	6.65

**FIGURE 6:**
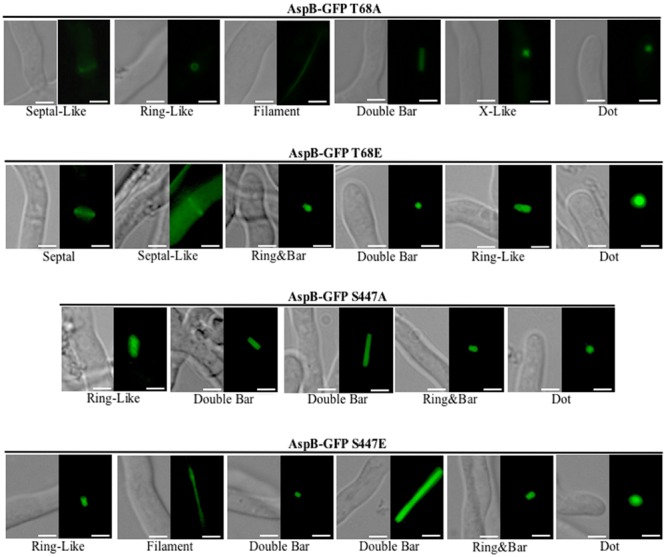
**AspB localization patterns in different AspB phosphomutant strains.** Phosphorylation and dephosphorylation of T68 and S447 are required for proper localization of AspB under normal growth conditions during the multicellular growth stage. AspB–EGFP phosphomimetic (glutamic acid substitutions) or non-phosphorylatable (alanine substitutions) strains were grown in GMM broth for 20 h and AspB–EGFP localization visualized by fluorescent microscopy. Scale bar, 2.5 μm.

To assess the role of these phosphorylated residues as possible posttranslational regulation sites, we next generated two non-phosphorylatable as well as two phosphomimetic mutant strains (T68A and T68E; S447A and S447E). Phenotypic analyses of these phosphomutant strains revealed that locking AspB in either a phosphorylation-mimic or non-phosphorylable state resulted in mislocalization of AspB (**Figure 6**). Similar to the wild-type and the *ΔparA* strains, AspB phosphomutants localized into rings and double bars. However, the length of the bars observed was different in each of the AspB phosphomutants, while the ring-like structures were similar to those found in the *ΔparA* strain. Although all four AspB phosphomutants showed the localization of AspB to dot-like structures associated with the hyphal tip, substitution of the serine or threonine to alanine resulted in a smaller dot structure compared to the dot structure in the glutamic acid substituted strain. AspB*^mt^*-T68E, AspB*^mt^*-S447A, and AspB*^mt^*-S447E localized in ring and bar structures, which is a localization pattern not observed in the *ΔparA* strain. AspB*^mt^*-T68A and AspB*^mt^*-T68E have septal-like localization, and only AspB*^mt^*-T68E localized transiently at septum. AspB*^mt^*-T68E and AspB*^mt^*-S447A did not abolish the AspB filament localization pattern. Similar to the *ΔparA* strain, each of these structures are fairly common across the respective AspB phosphomutant mycelia. However, mutation of these phosphorylation sites did not impact hyphal radial growth or conidiation (Supplementary Figures S2A,B). Additionally, the AspB*^mt^*-T68E strain had a 1.5-fold increase in interseptal distances when compared to the AspB–EGFP strain (*p* < 0.001; Supplementary Figure S2C).

## Discussion

We previously demonstrated that the *A. fumigatus* core septin AspB is required for critical cellular processes, including regular septation, conidiation, and conidial cell wall organization (Vargas-Muniz et al., 2015); however, regulation of AspB remains unclear. In *S. cerevisiae*, phosphorylation has been described as a possible mechanism of septin regulation (Tang and Reed, 2002; Garcia et al., 2011). Here, we explored the role of Gin4 and Cla4 kinases and the regulatory subunit of protein phosphatase 2A, ParA, as possible regulators of AspB. We found that AspB is phosphorylated at seven residues *in vivo* at the multicellular growth stage. Previously, core septins have been reported to be generally phosphorylated in the divergent amino- and carboxyl-termini, with the exception of *S. cerevisiae* Cdc10 (Hernandez-Rodriguez and Momany, 2012). However, most of the *A. fumigatus* AspB phosphorylation sites are within the GTPase domain. This is interesting because the majority of the known phosphorylation sites in septins are not present in the GTPase domain. While these residues are conserved across the filamentous ascomycetes, the same is not the case in other species. Due to the scattered pattern of septin phosphorylations observed in other species we expect no conservation in the residues phosphorylated (Hernandez-Rodriguez and Momany, 2012). However, further phosphoproteomic characterization is required to determine if phosphorylation of these residues is a conserved mechanism in other filamentous ascomycetes.

*Aspergillus fumigatus* Gin4 kinase is required for maintenance of proper interseptal distances and conidiation, similar to AspB. Deletion of *aspB* in the Δ*gin4* background exhibited a similar phenotype to the respective single deletion strains, suggesting that the Gin4 kinase and AspB may coordinately regulate septation. Nonetheless, AspB phosphorylation remains unchanged in the Δ *gin4* strain. This suggests that: (i) AspB might recruit Gin4 to possible septation sites and Gin4 then regulates septation, (ii) Gin4 indirectly regulates AspB through the phosphorylation of another yet unidentified AspB interacting partner, or (iii) Gin4 regulates AspB in a kinase-independent manner. Previous studies in *S. cerevisiae* have suggested that septins are required for proper localization and function of Gin4, and Gin4 phosphorylates Shs1 (non-core septin) and has functions that are independent from its kinase activity (Barral et al., 1998; Carroll et al., 1998; Longtine et al., 1998; Mortensen et al., 2002; Asano et al., 2006). Gin4 has been shown to regulates septins in a cell-cycle dependent manner in *S. cerevisiae* (Mortensen et al., 2002). It is still possible that Gin4 contributes to septin phosphorylation; however, this would be in a low abundant subset of AspB that we were not able to detect. Additionally, *A. nidulans* septins form distinct complexes at different growth stages (Hernandez-Rodriguez et al., 2014). The results reported here only focus on the multicellular growth stage, and it is possible we missed key regulatory phosphorylation events in the other growth stages. While the Δ*aspB* strain exhibited hypervirulence in the *Galleria* model but had no effect on virulence in the murine model (Vargas-Muniz et al., 2015), the Δ*gin4* strain is hypervirulent in both the insect and murine model of invasive aspergillosis. This could be due to misregulation of septin function in general or a septin-independent role of Gin4.

Deletion of the gene encoding the Cla4 kinase resulted in a dramatic reduction in hyphal growth as well as loss of polarity and hyperbranching (data not shown). These phenotypes are similar to those previously described in fungal plant pathogens, suggesting a conserved role of Cla4 in regulating hyphal morphology (Leveleki et al., 2004; Li et al., 2004; Rolke and Tudzynski, 2008; Frieser et al., 2011; Tian et al., 2015). Although the apical compartment lengths are not different from that in the wild-type strain, the Δ*cla4* strain is hyperseptated in the basal hyphal region, suggesting that Cla4 might not have a direct role in septum formation but instead coordinate septum positioning. A similar phenotype was observed in *M. oryzae*, where the *chm1* deletion strain had normal hyphal morphology but the hyphal compartment was significantly reduced (Li et al., 2004). In *C. albicans*, deletion of *cla4* resulted in reduction in fungal burden and hyphal invasion in mouse kidneys, leading to a reduction in pathogenesis (Leberer et al., 1997). The *A. fumigatus* Δ*cla4* strain also showed decreased tissue invasion in the intranasal murine model of invasive aspergillosis, however; the Δ*cla4* strain remained as virulent as the *akuB*^KU80^ strain. The Δ*cla4* strain did not phenocopy the Δ*aspB* strain or alter the AspB phosphorylation profile. Nonetheless, the AspB localization pattern in the Δ*cla4* strain was altered. It is suggested that Cla4 is capable of regulating the actin cytoskeleton in some fungi, and Cla4 could have a similar role in *A. fumigatus* (Leveleki et al., 2004). Furthermore, previously we showed that septin localization is dependent on actin and microtubules, and one of the septins, AspE, interacts with actin and microtubules (Juvvadi et al., 2011b, 2013). Thus, this altered localization could be a result of mislocalization of actin due to the absence of the Cla4 kinase.

Similar to *A. nidulans, A. fumigatus* ParA is involved in hyphal growth, conidiation and septation (Zhong et al., 2014). The Δ*parA* strain was hypovirulent in the *G. mellonella* model; however, the survival and histopathological analyses of the Δ*parA* strain were similar to that of the wild-type strain in the murine model. This difference in virulence between the two models could be due to the immunosuppression used in the murine model to establish infection, compared to the immunocompetent invertebrate model with a rudimentary immune system. Therefore, ParA is dispensable for pathogenesis in an immunosuppressed mammalian host. The role of RTS1 (ParA ortholog) as a septin regulator has been previously explored in *S. cerevisiae*, where it coordinates the dynamics of the septin rings by Rts1-dependent dephosphorylation of Shs1 (Dobbelaere et al., 2003). In *A. fumigatus*, deletion of *parA* resulted in altered AspB localization and, most importantly, altered the phosphorylation profile of AspB revealing ParA-dependent dephosphorylation of AspB. AspB under normal growth conditions is phosphorylated in seven residues distributed across the GTPase domain and the C-terminal region. Deletion of *parA* resulted in two additional phosphorylation sites (T68 and S447) that are enriched sevenfold when compared to the control strain. Our preliminary studies on the AspB interactome using purification of the AspB–EGFP fusion protein using GFP-Trap affinity matrix and subsequent LC–MS/MS analysis revealed PP2A as one of the AspB interactants, suggesting that PP2A could be directly dephosphorylating these residues (data not shown). Although mutation of these sites (S447A, S447E, T68A, and T68E) altered the localization pattern of AspB, only apical compartment length was affected in the AspB*^mt^*-T68E. Interestingly, AspB*^mt^*-T68E localizes transiently at the septum, similar to wild-type AspB, and the defect in apical compartment length could be due to altered interaction with septation regulators due to locking AspB in a phosphomimetic state. T68 is in the analogous position to *A. gossypii* S91 that has also been reported to be phosphorylated *in vivo* (Meseroll et al., 2013). A phosphomimetic strain of S91 in *A. gossypii* also resulted in a modest phenotype with abnormal spore morphology; however, spores were viable and capable of germinating at the same rate as the wild type. More detailed characterization of these mutants, as well as phosphomutants of the other AspB phosphoresidues, will further our understanding on how AspB is regulated through phosphorylation and how these phosphorylation sites might affect AspB–protein interactions.

## Author Contributions

JV and WS conceived and designed research; JV, HR, and AR performed research; JV, HR, AR, and YA performed virulence and histopathology analyses; JV GW, ES, and MM acquired and analyzed the phosphoproteomic data. JV, PJ, and WJ wrote the paper; JV, HR, AR, GW, ES, MM, YA, PJ, and WS approved the final submission.

## Conflict of Interest Statement

The authors declare that the research was conducted in the absence of any commercial or financial relationships that could be construed as a potential conflict of interest.

## Footnotes

1 www.aspergillusgenome.org
