# Supplementary material for: Dephosphorylation of the Core Septin, AspB, in a Protein Phosphatase 2A-Dependent Manner Impacts Its Localization and Function in the Fungal Pathogen Aspergillus fumigatus
Source: Front Microbiol. 2016 Jun 22;7:997. doi: 10.3389/fmicb.2016.00997 (PMC4916205; doi:10.3389/fmicb.2016.00997)
Supplement: Supplementary file 1 [file Table_1.DOCX]

**Table S1: List of Primers Used in This Study**

| **Name** | **Sequence (5’-3’)** | **Direction** |
| --- | --- | --- |
| **For Gin4 Deletion**  Gin4-5’-F  Gin4-5’-pyrG-ovlp-R  Gin4-3’-pyrG-ovlp-F  Gin4-3’-R | GTCTTATAGTCAGCCACTTCAAGCCC  ATCCATAGGATCAGCAGGCAAGCCCGGACTAATGAA  ACACCACCTACCCTTTCTAGCATGCCTGGCTGTCATTTC  TCTGCCTGTTCTGGAGGAACTTGA | Forward  Reverse  Forward  Reverse |
| **For Cla4 Deletion**  Cla4-5’-F  Cla4-5’-pyrG-ovlp-R  Cla4-3’-pyrG-ovlp-F  Cla4-3’-R | GGCAGTCTTGTATCCCATACAAGC  ATCCATAGGATCAGCACTGCGACAGAGGTCAGTCAAAGTTC ACACCACCTACCCTTTTCTTTGCACAATCCACTGTGCCAGC  CCTGCCGTTTAGTCAGGAAATACTCC | Forward  Reverse  Forward  Reverse |
| **For ParA Deletion**  ParA-promo-F-SalI  ParA-promo-R-EcoRI  ParA-term-F-BamHI  ParA-term-R-NotI | GATTGTCGACCAGTCTGAGCAATCAGATCTGG  GATTGAATTCTGTCAAGACTATGTAACCAGCC  GTTTGGATCCTTCTAACGTTGAAACGTCCG  GTTTGCGGCCGCCTCGACGAAAACGCTCTAGCTA | Forward  Reverse  Forward  Reverse |
| **For S447A Substitution**  AspB-gene-KpnI-F  AspB-S447A-R  AspB-S447A-F  GFP-R-NotI | GATTGGTACCTGTAGGATCGAAGCTCGAGGTTTCA  CTTCCAGCCTCGAGTCGGGCCTTCTT  AAGAAGGCCCGACTCGAGGCTGGAAG  GTTTCGCGGCCGCTTTACTTGTACAGCTCGTCCAT | Forward  Reverse  Forward  Reverse |
| **For S447E Substitution**  AspB-gene-KpnI-F  AspB-S447E-R  AspB-S447E-F  GFP-R-NotI | GATTGGTACCTGTAGGATCGAAGCTCGAGGTTTCA  CTTCCCTCCTCGAGTCGGGCCTTCTT  AAGAAGGCCCGACTCGAGGAGGGAAG  GTTTCGCGGCCGCTTTACTTGTACAGCTCGTCCAT | Forward  Reverse  Forward  Reverse |
| **For T68A Substitution**  AspB-gDNA-F-KpnI  AspB-T68A-R  AspB-T68A-F  GFP-R-NotI | GATTGGTACCATGGGTAAGCCCGCTTTCGATA  TAGCCAGCCAGTTTGCGGCGAA  TTCGCCGCAAACTGGCTGGCTA  GTTTCGCGGCCGCTTTACTTGTACAGCTCGTCCAT | Forward  Reverse  Forward  Reverse |
| **For T68E Substitution**  AspB-gDNA-F-KpnI  AspB-T68E-R  AspB-T68E-F  GFP-R-NotI | GATTGGTACCATGGGTAAGCCCGCTTTCGATA  TAGCCCTCCAGTTTGCGGCGAA  TTCGCCGCAAACTGGAGGGCTA  GTTTCGCGGCCGCTTTACTTGTACAGCTCGTCCAT | Forward  Reverse  Forward  Reverse |
