# Supplementary figures and images for: Dephosphorylation of the Core Septin, AspB, in a Protein Phosphatase 2A-Dependent Manner Impacts Its Localization and Function in the Fungal Pathogen Aspergillus fumigatus
Source: Front Microbiol. 2016 Jun 22;7:997. doi: 10.3389/fmicb.2016.00997 (PMC4916205; doi:10.3389/fmicb.2016.00997)

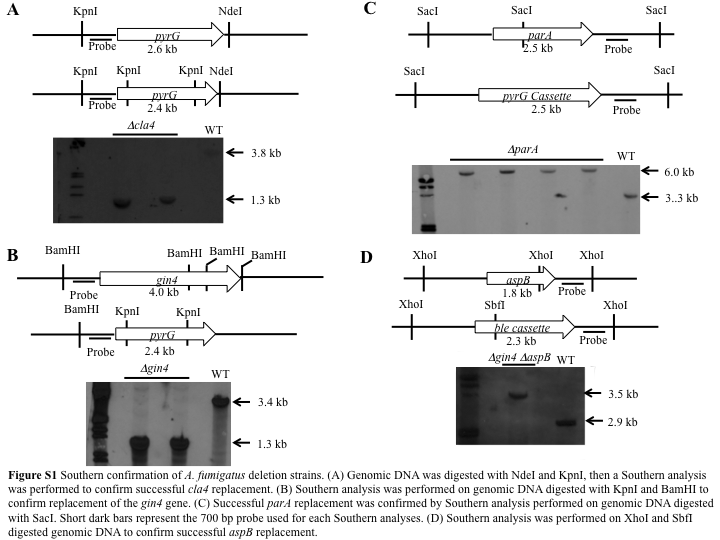

Supplement: Supplementary file 2 [file Image_1.TIFF]

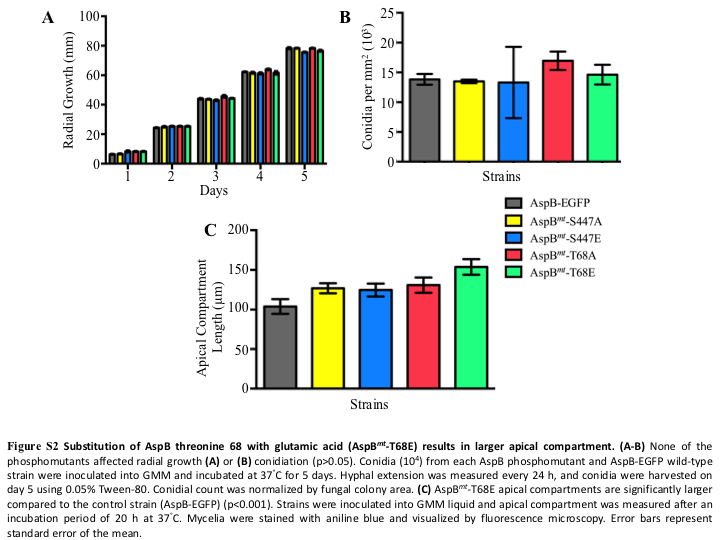

Supplement: Supplementary file 3 [file Image_2.TIFF]
